# Association Between Gastroesophageal Reflux Disease and Extraesophageal Malignancies: A Systematic Review and Meta-Analysis

**DOI:** 10.3390/cancers17233881

**Published:** 2025-12-04

**Authors:** Yu-Si Xu, Zhe-Ran Chen, Yan Bian, Ye Gao, Lei Xin, Luo-Wei Wang

**Affiliations:** Department of Gastroenterology, Changhai Hospital, Naval Medical University, Shanghai 200433, Chinagaoye@smmu.edu.cn (Y.G.)

**Keywords:** gastroesophageal reflux disease, GERD, epidemiology, systematic review, oncology

## Abstract

Background/Objectives: The association between gastroesophageal reflux disease (GERD) and extraesophageal malignancies remains unclear. This study aimed to systematically evaluate the relationship between GERD and these cancers. Methods: PubMed, Embase, Scopus, Cochrane Library, and Web of Science were searched for studies reporting risk estimates—risk ratios (RRs), odds ratios (ORs), hazard ratios (HRs), or standardized incidence ratios (SIRs)—of GERD and extraesophageal malignancies. Mendelian randomization (MR) studies with available risk estimates and 95% confidence intervals (CIs) were also included. Pooled estimates with 95% CIs were calculated using a random-effects model. Results: As of 21 May 2025, a total of 37 studies were included in the analysis. GERD was significantly associated with an increased risk of several extraesophageal malignancies, including pharyngeal cancer (pooled RR = 2.04; 95% CI: 1.38–3.02), with a particularly high risk observed for hypopharyngeal cancer (RR = 2.95; 95% CI: 1.99–4.37; I^2^ = 60.24%). Elevated risks were also identified for laryngeal cancer (RR = 2.23; 95% CI: 1.41–3.52) and lung cancer (RR = 1.20; 95% CI: 1.01–1.42). No significant association was found between GERD and colorectal cancer (RR = 1.19; 95% CI: 0.68–2.09), and findings regarding oral cancer were inconsistent across studies. Six MR studies confirmed a positive causal relationship between GERD and lung cancer, while one MR study suggested a potential causal association with oral cancer and another with pancreatic cancer. Conclusions: Our findings suggest that GERD may be a significant risk factor for pharyngeal, laryngeal and lung cancers. Appropriate evaluation and surveillance in patients with GERD may be warranted.

## Simple Summary

Gastroesophageal reflux disease (GERD) has been linked to esophageal cancer, but its association with extraesophageal malignancies remains unclear. Clarifying these risks may inform clinical surveillance strategies. This systematic review and meta-analysis found significant associations between GERD and increased risks of pharyngeal, laryngeal, and lung cancers, with Mendelian randomization supporting a potential causal relationship to lung cancer. The potential extraesophageal malignancies risks associated with GERD highlight the need for greater awareness and may support the development of targeted risk assessment or monitoring approaches.

## 1. Introduction

Gastroesophageal reflux disease (GERD) is one of the most common chronic gastrointestinal disorders, affecting nearly 783.95 million individuals worldwide [1]. It is characterized by esophageal mucosal damage or troublesome symptoms caused by the reflux of gastric contents into the esophagus [2,3,4], leading not only to impaired quality of life but also to substantial long-term health consequences [5]. The carcinogenic potential of chronic reflux has been well established in the esophagus, where GERD represents a principal risk factor for Barrett’s esophagus and subsequently esophageal adenocarcinoma [6,7]. However, whether GERD contributes to malignant transformation beyond the esophagus remains less clear.

In recent years, increasing attention has been directed toward possible links between GERD and extraesophageal malignancies [8,9,10]. Several observational studies have suggested elevated risks of cancers of the larynx [11,12], pharynx [9,10,13], oral cavity [14], and lung [15] among individuals with GERD, raising concerns about the systemic or upper aerodigestive carcinogenic effects of chronic reflux exposure. Despite these indications, findings across studies have been inconsistent, with substantial heterogeneity in study design, diagnostic definitions of GERD, and adjustment for confounders such as smoking and alcohol drinking. As a result, the magnitude, robustness, and clinical relevance of these associations remain uncertain.

Therapeutic management of GERD includes lifestyle modification, pharmacologic therapy—primarily proton pump inhibitors (PPIs)—and surgical interventions such as laparoscopic fundoplication [5]. These treatments effectively reduce esophageal acid exposure and alleviate symptoms, and long-term PPI therapy has been suggested to mitigate progression to Barrett’s esophagus and esophageal adenocarcinoma [16]. However, whether GERD treatment reduces the incidence of extraesophageal malignancies remains unknown. Existing evidence is limited. Therefore, determining whether GERD is associated with an increased risk of extraesophageal cancers is critical for understanding its broader clinical consequences.

To date, no comprehensive synthesis has systematically evaluated this potential relationship across all reported cancer sites. Clarifying whether GERD is associated with increased risks of extraesophageal malignancies is essential for understanding the broader implications of reflux disease, identifying high-risk populations, and informing future surveillance or prevention strategies.

Therefore, we conducted a systematic review and meta-analysis to assess the association between GERD and the risk of extraesophageal malignancies and to further explore the potential cause-and-effect relationship between GERD and these cancers, thereby providing more robust evidence regarding the long-term impact of GERD.

## 2. Materials and Methods

This systematic review and meta-analysis of observational studies was conducted in accordance with the Preferred Reporting Items for Systematic Reviews and Meta Analyses (PRISMA) guidelines [17]. The protocol for this study was registered in PROSPERO International Prospective Register of Systematic Reviews (registration number: CRD42024504172).

### 2.1. Search Strategy

A systematic search was conducted in the PubMed, Embase, Scopus, Cochrane Library, and Web of Science databases. The search was conducted on 21 May 2025. Search terms such as “gastroesophageal reflux”, “GERD”, “non-erosive reflux disease”, “NERD”, “erosive esophagitis”, “neoplasm”, “cancer”, and “malignancy” were used across all databases. The full search strategy is provided in the Supplemental Material. Additionally, studies referenced in the reference lists of the identified sources were also screened.

### 2.2. Study Outcomes and Eligibility Criteria

This systematic review and meta-analysis focused on two outcomes: (1) to examine the observational association between GERD and the risk of developing extraesophageal malignancies, and (2) to evaluate the potential causal relationship between GERD and extraesophageal malignancies through meta-analyses of Mendelian randomization (MR) analyses.

Titles and abstracts of all identified records were independently screened by two investigators. Full-text was reviewed for relevance, and any disagreements were resolved through discussion with a third investigator. Studies were included if they met the following criteria: (1) Studies reporting risk estimates (odds ratios [ORs], hazard ratios [HRs], risk ratios [RRs], or standardized incidence ratios [SIRs]) assessing the association between GERD and extraesophageal malignancies, or from which such estimates could be derived. All included cancer diagnoses were required to be confirmed by a gold-standard method, such as biopsy, or identified through medical records. GERD was diagnosed based on 24 h pH monitoring, endoscopy, clinical diagnosis, symptom-based questionnaires, or medical records. (2) MR studies that investigated the causal relationship between GERD and extraesophageal malignancies, with available risk estimates and corresponding 95% confidence intervals (CIs).

Case reports, reviews, letters, editorial comments, studies without available full text, and those unrelated to the research topic were excluded. In addition, studies focusing on esophageal cancer or gastric cardia adenocarcinoma, as well as those lacking data on individual cancer types, were excluded.

### 2.3. Data Extraction and Quality Assessment

Information was extracted independently by two investigators, and any discrepancies were resolved through discussion with a third investigator. For the first outcome, the following data were extracted: first author, year of publication, country, study design, participant characteristics (e.g., age, sex, smoking status, alcohol consumption), cancer subtype, the number of GERD cases and total sample size, and the definitions used for GERD and cancer. The number of cases and controls, risk estimates (OR, RR, HR, or SIR) with 95% CIs, and covariate adjustments were also collected. When multiple results were reported, the most fully adjusted estimates were used. Additional details, such as data sources, number of single-nucleotide polymorphisms (SNPs), and effect estimates with 95% CI, were extracted for the second outcome. The quality of studies included for the first outcome was evaluated using the Newcastle–Ottawa Scale (NOS) [18], with studies receiving ≥7 stars considered high quality.

### 2.4. Statistical Analysis

The first outcome was assessed using pooled RRs with corresponding 95% CI for cohort studies, and pooled ORs with corresponding 95% CI for case–control and cross-sectional studies. All estimates were combined using a random-effects model. Studies reporting HRs and SIRs were converted into RRs because the incidence of cancer in the general population is low, following the methods described by Siristatidis and Qiao [19,20]. Heterogeneity across studies was assessed using the I^2^ statistic, with values of <25%, 25–49%, 50–74%, and ≥75% interpreted as no, low, moderate, and high heterogeneity, respectively [21]. Subgroup analyses were conducted based on cancer subsite, gender, age, definition of GERD and adjustments when available. Leave-one-out sensitivity analyses were performed to assess the robustness of the results when five or more studies were included. For outcomes with sufficient number of studies (n > 10), we further performed meta-regression analyses. Publication bias was evaluated using funnel plots and Egger’s test (if the number of included studies ≥ 10).

For the second outcome, IVW MR estimates were pooled for the primary analysis. Sensitivity analyses were performed using the weighted median and MR-Egger methods, following the approach described by Zamani [22]. All statistical analyses were conducted using R (version 4.4.3), and a *p*-value <0.05 was considered statistically significant.

## 3. Results

### 3.1. Search Results and Study Characteristics

The search strategy identified 62,475 records on 21 May 2025, of which 29,360 were excluded due to duplication. After screening titles and abstracts, 33,035 records were excluded. After full-text screening, a total of 37 studies were included in the final meta-analysis [8,9,10,11,12,13,14,15,23,24,25,26,27,28,29,30,31,32,33,34,35,36,37,38,39,40,41,42,43,44,45,46,47,48,49,50,51]. A detailed list of excluded studies along with reasons for exclusion is provided in in Supplemental Material, Table S1.

**Figure 1:**
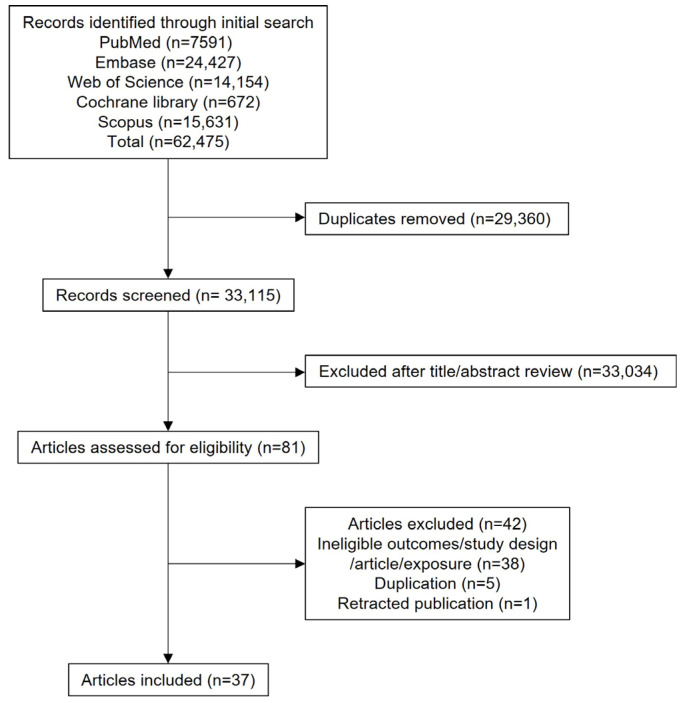
PRISMA flow diagram.

The characteristics of the included studies are summarized in Table 1 and Supplemental Material (Tables S2 and S3). Among the included studies, 29 were observational and 8 were MR studies. A flow diagram outlining the study selection process is shown in Figure 1.

### 3.2. Association Between GERD and Non-Esophageal Cancer

#### 3.2.1. Pharyngeal Cancer

**Figure 2:**
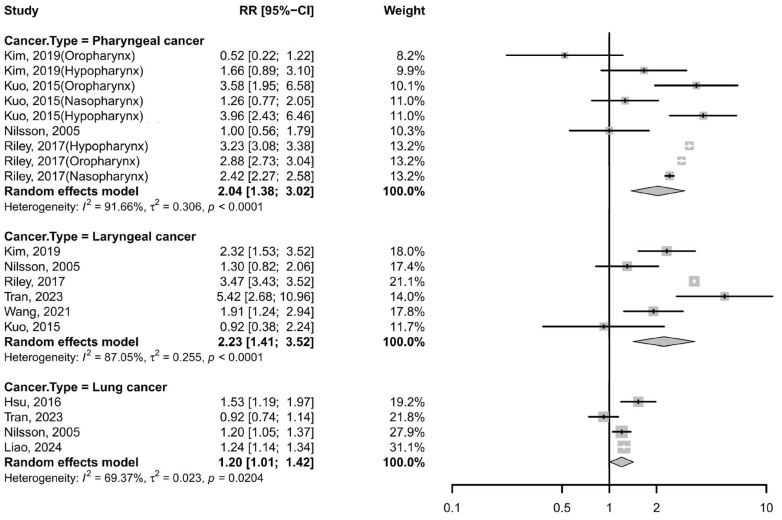
Forest plot of the risk of non-esophageal cancer among subjects with GERD cancer. CI, confidence interval; GERD, gastroesophageal reflux disease [8,9,10,13,15,32,34,46].

Nine studies (six cohort studies and three case–cohort studies) evaluated the association between GERD and pharyngeal cancer using risk estimates including RR, HR, SIR [8,9,10,13,14]. The pooled analysis yielded a RR of 2.04 (95% CI: 1.38–3.02; I^2^ = 91.66%; Figure 2), suggesting a significant positive association between GERD and pharyngeal cancer. Subgroup analysis by anatomical subsite revealed varying effects: the RR was 1.84 (95% CI: 0.59–5.72; *I*^2^ = 87.46%) for oropharyngeal cancer, 2.95 (95% CI: 1.99–4.37; *I*^2^ = 60.24%) for hypopharyngeal cancer, and 1.83 (95% CI: 0.97–3.45; *I*^2^ = 85.2%) for nasopharyngeal cancer. Despite stratification, high heterogeneity persisted across subgroups (Table S4). Sensitivity analyses confirmed that no single study substantially influenced the overall effect estimate.

**Figure 3:**
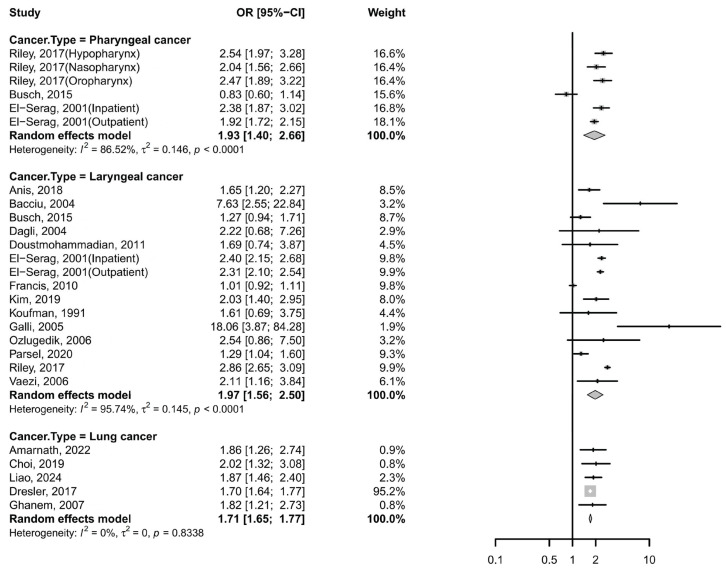
Forest plot of the risk of GERD among subjects with non-esophageal cancer. CI, confidence interval; GERD, gastroesophageal reflux disease [8,11,12,13,14,23,24,25,26,27,28,29,30,31,33,39,46,48,50].

Six case–control studies also supported this association between GERD and pharyngeal cancer [13,14,27,47], with a pooled OR of 1.93 (95% CI: 1.40–2.66; I^2^ = 86.52%, Figure 3). However, no significant associations were observed in subgroup analyses by cancer subtype: the pooled OR was 1.45 (95% CI: 0.44–4.84) for hypopharyngeal cancer and 1.45 (95% CI: 0.50–4.16) for oropharyngeal cancer. Despite stratification, high heterogeneity persisted across subgroups (Supplementary Table S4). Notably, sensitivity analysis indicated that omission of the study by Busch et al. [14] reduced heterogeneity substantially (*I*^2^ = 43.8%) and increased the pooled OR to 2.19 (95% CI: 1.93–2.50) (Table S5).

#### 3.2.2. Laryngeal Cancer

Five cohort studies and one case–cohort study reported RRs, HRs, or SIRs [8,9,10,13,32,34], with a pooled RR of 2.23 (95% CI: 1.41–3.52; I^2^ = 87.05%; Figure 2), indicating a significantly increased risk of laryngeal cancer among GERD patients. Subgroup analyses showed that the heterogeneity remained substantial across different stratifications (Supplementary Material Table S6). Sensitivity analyses confirmed that no single study substantially changed the overall effect estimate.

Additionally, fourteen case–control studies and one cross-sectional study consistently supported this association, with a pooled OR of 1.97 (95% CI: 1.56–2.50; I^2^ = 95.74%; Figure 3) [8,11,12,13,14,23,25,26,27,28,29,30,31,33]. The funnel plot appeared symmetrical (Figure S1), and Egger’s test indicated no significant publication bias (*p* = 0.8344). Despite stratification, high heterogeneity persisted across subgroups (Supplementary Table S6). Sensitivity analyses further confirmed the robustness of the findings, as no individual study substantially altered the pooled association. Meta-regression analyses indicated that sex, region, GERD diagnostic criteria, the number of participants and adjustments for smoking/drinking were significant contributors to the observed heterogeneity for laryngeal cancer (*p* < 0.05), whereas age showed less pronounced effects (Supplementary Table S7).

#### 3.2.3. Lung Cancer

Four cohort studies reported a pooled RR of 1.20 (95% CI: 1.01–1.42; I^2^ = 69.37%; Figure 2) [10,15,32,46], suggesting a modest but significant association. Notably, one of these studies, conducted by Liao et al. [46], identified positive associations between GERD and multiple subtypes—small cell lung cancer, squamous cell carcinoma, and adenocarcinoma. Removing Tran et al.’s study [32] reduced heterogeneity (*I*^2^ = 29.3%) and strengthened the association (RR 1.24, 95% CI: 1.17–1.33) (Table S5).

Five case–control studies reported a pooled OR of 1.71 (95% CI: 1.65–1.77; I^2^ = 0%; Figure 3) [24,39,46,48,50], indicating a significant association with no observed heterogeneity. Sensitivity analyses confirmed consistent results across all studies.

#### 3.2.4. Other Cancers

For colorectal cancer, three cohort studies reported no statistically significant association with GERD (pooled RR = 1.19, 95% CI: 0.68–2.09). Similarly, three studies investigating gastric (excluding gastric cardia) cancer showed a non-significant pooled estimate (RR: 1.57, 95% CI: 0.75–3.29) [45,49,51].

Findings for oral cancer were inconsistent across studies. Two cohort studies reported conflicting estimates (RR = 0.75, 95% CI: 0.43–1.31; vs. SIR = 1.33, 95% CI: 0.94–1.83) [8,9,32], and one case–control study conducted by Busch et al. reported an OR of 0.68 (95% CI: 0.46–1.02) [14], though all the result was not statistically significant.

Regarding other cancer types, Tran et al. found inverse associations between GERD and cancers of the liver and pancreatic cancers, but a positive association with thyroid cancer [32]. In another study, Riley reported a positive relationship between GERD and cancers of the tonsil and paranasal sinus [13]. However, these results are derived from single studies, limiting the ability to draw firm conclusions.

### 3.3. Cause-and-Effect Relationship Between GERD and Non-Esophageal Cancer

#### 3.3.1. Lung Cancer

**Figure 4:**
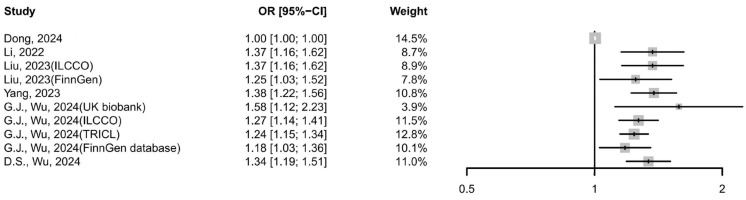
Forest plot of the cause-and-effect relationship between GERD and lung cancer using IVW methods. CI, confidence interval; GERD, gastroesophageal reflux disease [35,36,37,38,41,42].

Six MR studies [35,36,37,38,41,42] were included to assess the potential causal relationship between GERD and lung cancer. The meta-analysis using the IVW method revealed that a significant positive association (OR: 1.26 [95% CI:1.16–1.36], I^2^ = 93.55%, *p* <0.0001; Figure 4), with GERD increasing the risk of lung cancer, which was supported by the weighted median (1.00 [95% CI 1.00–1.01], I^2^ = 0%, *p* = 0.5280) and MR-Egger analyses (1.19 [95% CI 1.09–1.29], I^2^ = 82.52%, *p* < 0.0001). Leave-one-out analysis revealed that the study by Dong et al. [41] was a major source of heterogeneity; its exclusion reduced the I^2^ from 93.59% to 0%, likely due to its weak reported association (OR = 1.0027), narrow confidence interval, and disproportionate weight.

Subgroup analyses confirmed the causal relationship between GERD and all subtypes of lung cancer. The meta-IVW estimates showed a consistent positive association: 1.27 (95% CI: 1.16–1.38) for squamous cell carcinoma, 1.21 (95% CI: 1.12–1.30) for adenocarcinoma, and 1.87 (95% CI: 1.47–2.31) for small cell carcinoma. These findings were further supported by sensitivity analyses, with low heterogeneity observed across all subtypes (I^2^ = 0%).

#### 3.3.2. Other Cancers

One MR study conducted by Shen et al. reported a positive association between GERD and oral cavity cancer, with an IVW estimate of 1.90 (95% CI: 1.26–2.81) [40]. This finding was consistent with results from the weighted median (OR: 1.60 [95% CI: 0.70–3.70]) and MR-Egger methods (OR: 27.27 [95% CI: 0.31–290.04]).

In addition, one MR study reported a potential causal relationship between GERD and pancreatic cancer, with an IVW OR of 1.36 (95% CI: 1.04–1.80) [43]. The weighted median (OR: 1.28, 95% CI: 0.86–1.91) and MR-Egger (OR: 1.83, 95% CI: 0.37–9.09) results were consistent in direction, though not statistically significant.

## 4. Discussion

GERD is a chronic condition that necessitates long-term management. Current screening guidelines recognize chronic GERD as a risk factor for esophageal adenocarcinoma [52,53,54] and recommend appropriate clinical evaluation and surveillance for patients with additional risk factors, such as male sex or age ≥50 years. However, GERD is not currently considered a risk factor for extraesophageal malignancies. Our findings underscore the urgent need for increased clinical and public health attention to GERD in the context of extraesophageal cancer burden. Our meta-analysis revealed that GERD was significantly associated with an elevated risk of cancers of the pharynx, larynx, and lung, while no significant associations were found between GERD and either colorectal or gastric cancer.

The core pathophysiological feature of GERD is the retrograde movement of acidic and non-acidic gastric contents into the esophagus. When aspirated into adjacent or distant tissues, these refluxates—containing acid, pepsin, bile acids, and trypsin—may induce chronic mucosal injury. Biologically, this process is believed to contribute to carcinogenesis through mechanisms such as chronic inflammation, epithelial–mesenchymal transition, and angiogenesis [55,56]. This was supported by a prospective case–control study, in which a higher risk of laryngeal cancer was observed among patients who had undergone gastrectomy, suggesting a potential role of duodenal content (including bile acids and trypsin), pepsin, and acid residues on the development of laryngeal malignancies [57]. Moreover, the presence of pepsin in the airways of GERD patients at significantly higher levels than in controls was confirmed, further supporting the role of aspiration in carcinogenesis [58].

The pharynx—particularly the oropharynx—and larynx are anatomically adjacent to the esophagus and directly exposed to gastric refluxate, making them susceptible to injury from acid, pepsin, and bile acids. Chronic exposure of the upper aerodigestive tract to refluxate can induce epithelial inflammation, oxidative stress, and microerosion, predisposing mucosal tissue to malignant transformation. These biological processes may partially explain the elevated risks identified in our synthesis. Also, this anatomical proximity, together with the feasibility of endoscopic surveillance, highlights the clinical relevance of assessing cancer risk in these regions. Our meta-analysis revealed a significant association between GERD and laryngeal cancers, consistent with previous findings [31,59,60]. Although tobacco and alcohol use are established confounders [61,62], subgroup analyses adjusting for these factors showed only slightly attenuated effect estimates without statistical significance. Moreover, several studies demonstrated positive associations even among nonsmokers and nondrinkers, further supporting GERD as a potential independent risk factor for malignancies in the upper digestive tract [23,63].

Lung cancer is the most prevalent cancer worldwide. While previous studies have reported inconsistent findings regarding the association between GERD and lung cancer [32,39], our meta-analysis supports a positive association. Given that tobacco smoking is a major confounder, the included studies had adjusted for or excluded smoking as a variable, and the pooled OR estimates still demonstrated a positive association. MR analyses also support a causal relationship, with all IVW estimates demonstrating significant associations independent of smoking status. These findings are reinforced by a population-based cohort study, which found that anti-reflux surgery was associated with a reduced risk of small-cell and squamous-cell lung cancers [64].

Compared with previous meta-analyses exploring the relationship between GERD and laryngeal or pharyngeal cancer [59,65]—which included only case–control or cross-sectional studies and confirmed associations without addressing causality—our study incorporated cohort studies, thereby providing more robust evidence with long-term follow-up. This methodological improvement strengthens the reliability of the observed associations. Furthermore, to minimize publication bias and ensure comprehensive data inclusion, we also included eligible conference abstracts. Importantly, our integration of MR studies allowed us to assess potential causal relationships between GERD and non-esophageal cancers, offering insights beyond those achievable through traditional observational designs. To our knowledge, this is the most up-to-date and comprehensive synthesis of evidence on this topic.

Notably, there is no established guideline recommending surveillance solely because of GERD for most extraesophageal cancers. Routine cancer screening solely on the basis of GERD symptoms is not supported by the current evidence. Instead, a targeted, risk-stratified approach is more appropriate. Clinicians should consider intensified evaluation or referral for patients with GERD who present additional high-risk factors, such as older age, heavy smoking history, obesity, or a family history of relevant malignancy. For these patients, a lower threshold for diagnostic work-up and counseling on modifiable risk factors (smoking cessation, alcohol reduction, weight management) is reasonable. Also, while standard therapeutic protocols such as PPIs and lifestyle interventions effectively reduce esophageal inflammation and symptoms, evidence regarding their ability to lower the incidence of extraesophageal cancers is limited. Future studies should explore whether early intervention in GERD patients can modify cancer risk and identify subgroups who may benefit most from targeted prevention strategies.

One strength of our analysis is the incorporation of MR evidence, which helps mitigate key biases inherent to observational studies, such as confounding and reverse causation [66]. However, MR studies also have important limitations that must be acknowledged. Their validity depends heavily on the selection of robust genetic instruments. Although we ensured instrument strength by including variants with F-statistics >10, MR analyses may still be affected by horizontal pleiotropy, population stratification, and violations of the core instrumental variable assumptions. Moreover, the genetic architecture of GERD may differ across populations, potentially contributing to the observed heterogeneity among MR estimates.

This study has several limitations. First, the exclusion of studies that defined GERD based solely on symptoms may have led to an underestimation of the true associations, as some cases of GERD might have been missed. Second, substantial heterogeneity was observed in several analyses, which could not be fully accounted for through subgroup or sensitivity analyses, and may reflect differences in study populations, diagnostic criteria for GERD, and study designs. Lastly, for cancer types such as oral, thyroid, colorectal, gastric, and pancreatic cancers, the available evidence is limited and sometimes inconsistent. Therefore, while our meta-analysis did not identify statistically significant associations for these cancers, this should be interpreted cautiously, as the small number of studies prevents definitive conclusions. These limitations underscore the need for future high-quality, large-scale studies with standardized definitions of GERD and cancer outcomes to more accurately elucidate the relationship between GERD and non-esophageal cancers.

## 5. Conclusions

In summary, this systematic review and meta-analysis investigated the association between GERD and extraesophageal cancers. We found evidence of a positive association between GERD and certain malignancies, including pharyngeal, laryngeal, and lung cancer. Meta-analyses of MR studies suggested a potential causal link with lung cancer. However, for other cancer types, the evidence remains limited and inconclusive. These findings highlight the need for further high-quality studies before making definitive public health recommendations.

## Figures and Tables

**Table 1:** Characteristics of included observational studies.

Reference	Country	Study Design	Number of Participants ^a^	Assessment of GERD	**Quality ^b^**
Amarnath, 2022 [39]	U.S.	Retrospective case–control	1083 (543/540)	ICD 9/10 and endoscopy	7
Anis, 2018 [11]	U.S.	Retrospective case–control	2730 (290/2440)	ICD 9	6
Bacciu, 2004 [23]	Italy	Retrospective case–control	161 (36/125)	Endoscopy	7
Busch, 2016 [14]	U.S.	Retrospective case–control	2718 (1340/1378)	A history of medical diagnosis	8
Choi, 2019 [24]	Korea	Retrospective case–control	1070 (214/856)	ICD 10	7
Dağli, 2004 [25]	Turkey	Prospective case–control	47 (22/25)	24 h pH monitoring	4
Doustmohammadian, 2011 [26]	Iran	Prospective case–control	130 (65/65)	Endoscopy	7
Dresler, 2017 [50]	UK	Retrospective case–control	93616 (19,143/74,473)	PPI use for GERD	2
Durán, 2008 [47]	Mexico	Retrospective case–control	173 (43/130)	Endoscopy	6
El-Serag, 2001 [27]	U.S.	Retrospective case–control	Hospitalized: 41,140 (8228/32,912) [laryngeal cancer]; (1912/7648) [pharyngeal cancer]; Outpatient: 46,460 (9292/37,168) [laryngeal cancer]; 13,845 (2769/11,076) [pharyngeal cancer]	ICD-9-CM	5
Francis, 2011 [12]	U.S.	Retrospective case–control	28,898 (14,449/14,449)	ICD 9	5
Galli, 2002 [28]	U.S.	Prospective case–control	42 (21/21)	24 h pH monitoring	6
Ghanem, 2007 [48]	U.S.	Retrospective case–control	650 (325/325)	Endoscopy, manometry or clinical diagnosis	5
Hayasaka, 2024 [49]	Japan	Retrospective case–control	180 (20/160)	Endoscopy	7
Hsu, 2016 [15]	China	Retrospective cohort	76,378 (15,412/60,957)	ICD-9 cm and procedure codes for endoscopy	9
Hu, 2021 [51]	China	Retrospective cohort	274,968 (45,828/229,140)	ICD-9 cm and diagnosed by gastroenterologists (at least two consensus diagnoses)	9
Kim, 2019 [8]	Korea	I: retrospective cohort; II: retrospective case–control	I: 296,121 (98,707/197,414); II: 1115 (223/892)	ICD 10 that participants treated for GERD ≥ 2 times and prescribed a proton pump inhibitor (PPI) for ≥2 weeks	I: 7; II: 7
Koufman, 1991 [29]	U.S.	Prospective case–control	182 (31/151)	24 h pH monitoring	5
Kuo, 2015 [9]	China	Retrospective cohort	-(39,845/-)	ICD-9 cm and had been diagnosed based on results from endoscopy or a 24 h pH-metrics monitoring	9
Liao, 2024 [46]	England, Wales, and Scotland	I: Cross-sectional; II: prospective cohort	I: 501,569 (321/501,248); II: 458,691 (58,191/400,500)	Self-report, ICD 10/9 diagnosis, operative procedures linked to hospital inpatient records, and self-reported GERD-related medication use	II:7
Nilsson, 2005 [10]	Sweden	Retrospective cohort	-(66,965/-)	Hospitalized esophagitis, hiatal hernia, and/or heartburn	7
Ozlugedik, 2006 [30]	Turkey	Prospective case–control	62 (29/33)	24 h pH monitoring	5
Parsel, 2020 [31]	U.S.	Retrospective case–control	2094 (698/1396)	ICD 10	6
Riley, 2017 [13]	U.S.	I: Retrospective case–control; II: case–cohort	I: 16,694 (8347/8347) [laryngeal cancer]; 1496 (748/748) [hypopharyngeal cancer]; 14,448 (724/724) [oropharyngeal cancer]; 3970 (1985/1985) [tonsil cancer]; 1510 (755/755) [nasopharyngeal cancer]; 2492 (1246/1246) [paranasal sinus cancer];	ICD-9-CM	7
Solaymani-Dodaran, 2004 [45]	UK	Prospective cohort	19,808 (6392/13,416)	Endoscopy	8
Tran, 2023 [32]	Korea	Retrospective cohort	41,044 (10,261/30,783)	Individuals recorded with GERD (ICD-10) at least twice and treated for GERD with PPI or H2RA for at least 8 weeks as GERD patients	7
Vaezi, 2006 [33]	U.S.	Prospective case–control	288 (96/192)	Symptomatic GERD, ICD-9 codes, and long-term acid-suppressive therapy	7
Wang, 2021 [34]	U.S.	Retrospective cohort	490,605 (116,476/374,129)	Two or more Medicare claims from Medicare dataset	8
Wu, 2003 [44]	U.S.	Retrospective case–control	1798 (443/1355)	In-person interview and structured questionnaire (e.g., ever diagnosed by a physician)	8

CI, confidence interval; ICD, International Classification of Diseases; NSCLC, non-small cell lung cancer; ICD-9-CM, the 9th revision of the Clinical Modification of International Classification of Diseases; GERD, gastroesophageal reflux disease. ^a^ Numbers in parentheses represented number of participants in GERD/control group (cohort studies), or number of cases/controls (case–control studies); ^b^ Study quality was assessed based on the Newcastle–Ottawa Scale (range, 0–9 stars), details see Supplemental Material A and B.

## Data Availability

The data supporting the conclusions of this article is included within the article and its Supplemental Material.

## Supplementary Materials

The following supporting information can be downloaded at: https://www.mdpi.com/article/10.3390/cancers17233881/s1, Table S1. Table of excluded studies with rationale; Table S2. Baseline characteristics of the Mendelian randomization studies assessing the relationship of GERD and cancer; Table S3. Baseline characteristics of the Mendelian randomization studies assessing the relationship of GERD and lung cancer on specific subtype; Table S4. Subgroup analysis results assessing the relationship of GERD and pharyngeal cancer; Table S5. Leave-one-out meta-analysis results; Table S6. Subgroup analysis results assessing the relationship of GERD and laryngeal cancer; Table S7. Meta-regression results; Figure S1. Funnel plot to assess publication bias across the studies evaluating the risk of GERD among subjects with laryngeal cancer.

## Abbreviations

The following abbreviations are used in this manuscript:

GERD	Gastroesophageal Reflux Disease
RR	Risk Ratio
OR	Odds Ratios
HR	Hazard Ratios
MR	Mendelian Randomization
CI	Confidence Intervals
SIR	Standardized Incidence Ratio
