# Supplementary material for: Association Between Gastroesophageal Reflux Disease and Extraesophageal Malignancies: A Systematic Review and Meta-Analysis
Source: Cancers (Basel). 2025 Dec 4;17(23):3881. doi: 10.3390/cancers17233881 (PMC12691378; doi:10.3390/cancers17233881)
Supplement: Supplementary file 1 [file cancers-17-03881-s001.zip › cancers-4002715-supplementary.pdf]

Supplementary Material

Yu-Si Xu et al. Association Between Gastroesophageal Reflux Disease and Extraesophageal Malignancies: A Systematic Review and Meta-Analysis

Contents

Search strategy 2

- **Search strategy for Embase** 2
- **Search strategy for PubMed** 2
- **Search strategy for Scopus** 2
- **Search strategy for Web of Science** 3
- **Search strategy for Cochrane Library** 3

Table S1. Table of excluded studies with rationale 5

Table S2. Baseline characteristics of the Mendelian randomization studies assessing the relationship of GERD and cancer 6

Table S3. Baseline characteristics of the Mendelian randomization studies assessing the relationship of GERD and lung cancer on specific subtype 8

Table S4. Subgroup analysis results assessing the relationship of GERD and pharyngeal cancer 9

Table S5. Leave-one-out meta-analysis results 11

Table S6. Subgroup analysis results assessing the relationship of GERD and laryngeal cancer 12

Table S7. Meta-regression results 14

Figure S1. Funnel plot to assess publication bias across the studies evaluating the risk of GERD among subjects with laryngeal cancer 15

Material A. Study quality assessment for cohort studies (Newcastle-Ottawa Scale) 16

Material B. Study quality assessment for case-control studies (Newcastle-Ottawa Scale) 17

PRISMA Checklist 18

Search strategy

■ Search strategy for Embase

No.	Query	Search results (21 May 2025)
#1	'gastroesophageal reflux'/exp OR 'non erosive reflux disease'/exp OR 'reflux esophagitis'/exp OR 'acid reflux':ab,kw,ti OR 'gastroesophageal reflux':ab,kw,ti OR GERD:ab,kw,ti OR 'esophageal reflux':ab,kw,ti OR ' reflux esophagitis ':ab,kw,ti OR NERD:ab,kw,ti OR 'non-erosive reflux':ab,kw,ti OR 'erosive esophagitis':ab,kw,ti	97,423
#2	'neoplasm'/exp OR neoplasm*:ab,kw,ti OR cancer*:ab,kw,ti OR carcinoma*:ab,kw,ti OR malignan*:ab,kw,ti OR tumor*:ab,kw,ti OR tumour*:ab,kw,ti OR adenocarcinoma*:ab,kw,ti OR sarcoma*:ab,kw,ti OR lymphoma*:ab,kw,ti	7,904,109
#3	#1 AND #2	24,427

■ Search strategy for PubMed

("gastroesophageal reflux"[MeSH Terms] OR "gastroesophageal reflux"[Title/Abstract] OR GERD[Title/Abstract] OR "acid reflux"[Title/Abstract] OR "reflux esophagitis"[Title/Abstract] OR "Non-Erosive Reflux Disease"[Title/Abstract] OR "NERD"[Title/Abstract] OR "erosive esophagitis"[Title/Abstract] OR "esophageal reflux"[Title/Abstract])
AND
("neoplasm"[MeSH Terms] OR neoplasm*[Title/Abstract] OR cancer*[Title/Abstract] OR carcinoma*[Title/Abstract] OR malignan*[Title/Abstract] OR tumor*[Title/Abstract] OR tumour*[Title/Abstract] OR adenocarcinoma*[Title/Abstract] OR sarcoma*[Title/Abstract] OR lymphoma*[Title/Abstract])
Search results (21 May 2025): 7,591 Results.

■ Search strategy for Scopus

No.	Query
**#1**	TITLE-ABS-KEY (“gastroesophageal reflux” OR “acid reflux” OR “ esophageal reflux “ OR “reflux esophagitis” OR GERD OR "non-erosivereflux " OR NERD OR "erosive esophagitis")
**#2**	TITLE-ABS-KEY (neoplasm* OR cancer* OR carcinoma* OR malignan* OR tumor* OR tumour* OR adenocarcinoma* OR sarcoma* OR lymphoma*)
**#3**	#1 AND #2
Search results (21 May 2025): 15,631 Results.	

■ **Search strategy for Web of Science**

No.	Query
#1	TS= ("Gastroesophageal Reflux” OR “gastric acid reflux” OR GERD OR “esophageal reflux” OR “reflux esophagitis” OR “Non-Erosive Reflux” OR NERD OR “erosive esophagitis”)
#2	TS= (neoplasm* OR cancer* OR carcinoma* OR malignan* OR tumor* OR tumour* OR adenocarcinoma* OR sarcoma* OR lymphoma*)
#3	#1 AND #2
Search results (21 May 2025): 14,154 Results.	

■ **Search strategy for Cochrane Library**

No.	Query	Search results (21 May 2025)
#1	MeSH descriptor: [Gastroesophageal Reflux] explode all trees	2,556
#2	(Gastroesophageal Reflux OR acid reflux OR GERD OR esophageal reflux OR reflux esophagitis OR Non-Erosive Reflux OR NERD OR erosive esophagitis):ti,ab,kw	7,190
#3	MeSH descriptor: [Neoplasms] explode all trees	127,284
#4	(neoplasm* OR cancer* OR carcinoma* OR malignan* OR tumor*	296,660

	OR tumour* OR adenocarcinoma* OR sarcoma* OR lymphoma*):ti,ab,kw	
#5	#1 OR #2	7,254
#6	#3 OR #4	306,261
#7	#5 AND #6	672

Table S1. Table of excluded studies with rationale

**Reason for exclusion (Number)**
Ineligible outcomes/study design/article type/exposure (n=38)
Duplication/same data source (n=5)
Retracted publication (n=1)

Table S2. Baseline characteristics of the Mendelian randomization studies assessing the relationship of GERD and cancer

Study	Data source or Country	Total population (n)	Cancer cases (n)	SNPs (n)	IVW-random (95%CI)	Weighted median (95%CI)	MR-Egger (95%CI)	Cancer type
Dong, 2024[35]	UK Biobank	374,687	2,671	65[a]	1.0027 (1.0012-1.0043)	1.0036 (1.0014-1.0057)	1.004 (0.996-1.013)	Lung
Li, 2022[36]	ILCCO	27,209	11,348	67 [a]	1.37 (1.16-1.63)	1.33 (1.08-1.64)	1.48 (0.57-3.83)	Lung
Liu, 2023[37]	ILCCO	27,209	11,348	77 [b]	1.37 (1.16-1.62)	1.32 (1.06-1.62)	1.94 (0.73-5.14)	Lung
Liu, 2023[37]	FinnGen database	242,708	4,030	76 [b]	1.25 (1.03-1.52)	1.09 (0.85-1.39)	1.16 (0.37-3.62)	Lung
Yang, 2023[38]	ILCCO, LC3	85,716	29,266	72 [a]	1.38 (1.22-1.56)	1.27 (1.10-1.47)	1.00 (0.51-1.96)	Lung
G.J., Wu, 2024[42]	UK biobank	456,348	535	199 [d]	1.58 (1.12-2.24)	1.49 (0.92-2.42)	3.46 (0.54-22.1)	Lung
G.J., Wu, 2024[42]	ILCCO	27,209	11,348	192 [d]	1.27 (1.14-1.41)	1.29 (1.11-1.5)	1.54 (0.87-2.73)	Lung
G.J., Wu, 2024[42]	TRICL	85,449	29,863	198 [d]	1.24 (1.15-1.34)	1.2 (1.08-1.32)	1.06 (0.71-1.59)	Lung
G.J., Wu, 2024[42]	FinnGen database	2634,448	3,865	196 [d]	1.18 (1.03-1.36)	1.1 (0.91-1.33)	0.62 (0.3-1.31)	Lung
D.S., Wu, 2024[41]	ILCCO	85,716	29,266	87 [c]	1.34 (1.19-1.51)	1.28 (0.54-1.27)	1.08 (0.54-2.17)	Lung
Shen, 2025[40]	Europe, North America and South America	1,135	3,464	41 [a]	2.6 (1.47-4.59)	1.6 (0.7-3.7)	27.27 (0.31-290.04)	Oral cavity
Yang, 2025[38]	MRC IEU OpenGWAS	476,245	1,196	76 [a]	1.36 (1.04-1.80)	1.28 (0.86,1.91)	1.83 (0.37-9.09)	Pancreas

Abbreviations: GERD, gastro-esophageal reflux disease; OR, odds ratio; ILCCO, International Lung Cancer Consortium; LC3, Lung Cancer Cohort Consortium; TRICL, Transdisciplinary Research in Cancer of the Lung; IVW, inverse variance weighted.

a. Selected based on genome-wide significance ($p < 5\times10^{-8}$) and LD clumping ($r^2 < 0.001$), F>10

b. Selected based on genome-wide significance ($p < 5\times10^{-8}$) and LD clumping ($r^2 > 0.01$), F>10
c. Selected based on genome-wide significance ($p < 5\times10^{-8}$) and LD clumping ($r^2 = 0.01$), F>10
d. Selected based on genome-wide significance ($p < 5\times10^{-8}$) and LD clumping ($r^2 < 0.01$), F>10

Table S3. Baseline characteristics of the Mendelian randomization studies assessing the relationship of GERD and lung cancer on specific subtype

Study	Data source or Country	IVW-random (95%CI)	Weighted median (95%CI)	MR-Egger (95%CI)	Cancer subtype
Li, 2022[36]	ILCCO	1.56 (1.26-1.93)	1.23 (0.92-1.65)	1.34 (0.41-4.41)	Squamous cell carcinoma
Li, 2022[36]	ILCCO	1.27 (1.01-1.60)	1.27 (0.93-1.70)	0.61 (0.17-2.10)	Adenocarcinoma
Liu, 2023[37]	ILCCO, FinnGen	1.27 (1.02-1.59)	1.28 (0.94-1.72)	0.8 (0.23-3.09)	Adenocarcinoma
Liu, 2023[37]	ILCCO, FinnGen	1.5 (1.22-1.86)	1.25 (0.92-1.69)	2.21 (0.63-7.74)	Squamous cell carcinomas
Liu, 2023[37]	ILCCO, FinnGen	2.15 (0.87-5.31)	1.13 (0.54-2.37)	0.59 (0.03-13.57)	Small cell carcinoma
Yang, 2023[38]	ILCCO, LC3	1.53 (1.31-1.79)	1.36 (1.11-1.68)	0.84 (0.36-1.99)	Squamous cell carcinoma
Yang, 2023[38]	ILCCO, LC3	1.20 (1.03-1.40)	1.12 (0.93-1.36)	0.71 (0.31-1.63)	Adenocarcinoma
Yang, 2023[38]	ILCCO, LC3	1.79 (1.36-2.35)	1.99 (1.40-2.83)	6.08 (1.30-28.34)	Small cell carcinoma
G.J., Wu, 2024[42]	FinnGen	1.19 (0.95-1.49)	1.23 (0.89-1.70)	1.58 (0.48-5.19)	Adenocarcinoma
G.J., Wu, 2024[42]	ILCCO	1.12 (0.96-1.30)	1.14 (0.91-1.42)	1.19 (0.55-2.59)	Adenocarcinoma
G.J., Wu, 2024[42]	TRICL	1.15 (1.05-1.26)	1.16 (1.02-1.32)	1.03 (0.64-1.66)	Adenocarcinoma
G.J., Wu, 2024[42]	FinnGen	1.20 (0.93-1.54)	1.02 (0.73-1.42)	0.65 (0.17-2.46)	Squamous cell carcinoma
G.J., Wu, 2024[42]	ILCCO	1.32 (1.13-1.54)	1.16 (0.92-1.46)	1.37 (0.61-3.04)	Squamous cell carcinoma
G.J., Wu, 2024[42]	TRICL	1.35 (1.20-1.51)	1.28 (1.10-1.50)	1.00 (0.55-1.82)	Squamous cell carcinoma
D.S., Wu, 2024[41]	ILCCO	1.52 (1.32-1.75)	1.39 (1.13-1.71)	0.93 (0.42-2.09)	Squamous cell carcinoma
D.S., Wu, 2024[41]	ILCCO	1.25 (1.08-1.45)	1.36 (1.15-1.61)	0.72 (0.31-1.67)	Adenocarcinoma
D.S., Wu, 2024[41]	ILCCO	1.76 (1.37-2.27)	1.89 (1.37-2.62)	5.84 (1.34-25.5)	Small cell carcinoma

Abbreviations: GERD, gastro-esophageal reflux disease; OR, odds ratio; ILCCO, International Lung Cancer Consortium; LC3, Lung Cancer Cohort Consortium; TRICL, Transdisciplinary Research in Cancer of the Lung; IVW, inverse variance weighted.

Table S4. Subgroup analysis results assessing the relationship of GERD and pharyngeal cancer

	Pharyngeal cancer							
All studies	n	RR (95%CI)	I^2(%)	*P*	n	OR (95%CI)	I^2(%)	*P*
**Definition of GERD**								
Endoscopy	-	-	-	-	-	-	-	-
ICD codes	9	2.04(1.38-3.02)	91.66	＜0.001	6	1.93(1.40-2.66）	86.52	＜0.0001
Multiple metrics	-	-	-	-	-	-	-	-
Questionnaire	-	-	-	-	-	-	-	-
24h pH metrics	-	-	-	-	-	-	-	-
**Gender**								
male≤50%	5	1.79(0.90-3.59)	83.58	＜0.001	-	-	-	-
male＞50%	-	-	-	-	6	1.93(1.40-2.66）	86.52	＜0.001
**Age**								
≤60	5	1.79(0.90-3.59)	83.58	＜0.001	1	1.92(1.72-2.15)	-	-
＞60	-	-	-	-	5	1.92(1.28-2.88)	89.06	＜0.001
**Geographical region**								
North America	3	2.83(2.40-3.33)	96.09	＜0.001	6	1.93(1.40-2.66）	86.52	＜0.001
Europe	1	1.00(0.56-1.79)	-	-	-	-	-	-
Asia	5	1.79(0.90-3.59)	83.58	＜0.001	-	-	-	-
**Number of participants**								
≤10000	3	2.83（2.40-3.33）	96.09	＜0.001	4	1.82（1.09-3.02）	91.29	＜0.001
＞10000	6	1.63（0.90-2.97）	82.79	＜0.001	2	2.08（1.70-2.55）	60.56	＜0.001
**Quality of study**								
Good	9	2.04(1.38-3.02)	91.66	＜0.001	4	1.82(1.09-3.02)	91.29	＜0.0001
Fair	-	-	-	-	2	2.08(1.70-2.55)	60.56	0. 1113

All studies	Pharyngeal cancer n	RR (95%CI)	I^2(%)	*P*	n	OR (95%CI)	I^2(%)	*P*
Poor								
**Adjustments**								
BMI								
Yes	-	-	-	-	1	0.83（0.60-1.14）	-	-
No	9	2.04(1.38-3.02)	91.66	＜0.001	5	2.19（1.93-2.50）	43.83	0.1296
**Alcohol consumption**								
Yes	1	1.00(0.56-1.79)	-	-	3	1.58（0.85-2.92）	93.01	＜0.001
No	8	2.23(1.51-3.29)	91.57	＜0.001	3	2.34（2.01-2.73）	0	0.4567
**Smoking status**								
Yes					3	1.58（0.85-2.92）	93.01	＜0.001
No	9	2.04(1.38-3.02)	91.66	＜0.001	3	2.34（2.01-2.73）	0	0.4567
**Age**								
Yes	2	0.96(0.31-3.00)	78.44	0.0313	6	1.93(1.40-2.66）	86.52	＜0.001
No	7	2.42(1.71-3.43)	92.24	＜0.001	−	-	-	-
**Sex**								
Yes	2	0.96(0.31-3.00)	78.44	0.0313	6	1.93(1.40-2.66）	86.52	＜0.001
No	7	2.42(1.71-3.43)	92.24	＜0.001	-	-	-	−

Table S5. Leave-one-out meta-analysis results

	Risk estimates(95%CI)	I² (%)
**Pharyngeal cancer**		
Omitting Riley, 2017(Hypopharynx) [13]	1.82 (1.25; 2.65)	87.9
Omitting Riley, 2017(Nasopharynx) [13]	1.90 (1.28; 2.82)	89.2
Omitting Riley, 2017(Oropharynx) [13]	1.83 (1.25; 2.67)	88.3
Omitting Busch, 2015 [14]	2.19 (1.93; 2.50)	43.8
Omitting El-Serag, 2001(Inpatient) [27]	1.84 (1.25; 2.71)	88.4
Omitting El-Serag, 2001(Outpatient) [27]	1.92 (1.28; 2.88)	89.1
**Lung cancer**		
Omitting Hsu, 2016 [15]	1.14 (0.97; 1.34)	68.9
Omitting Tran, 2023 [32]	1.25 (1.17; 1.33)	29.3
Omitting Nilsson, 2005 [10]	1.20 (0.92; 1.57)	79.5
Omitting Liao, 2024 [46]	1.18 (0.90; 1.55)	78.2

Abbreviations: CI, confidence interval.

Table S6. Subgroup analysis results assessing the relationship of GERD and laryngeal cancer

	Laryngeal cancer							
	n	RR(95%CI)	I^2(%)	*P*	n	OR(95%CI)	I^2(%)	*P*
**Definition of GERD**								
Endoscopy					2	3.43(0.79-15.01)	78.36	0.0316
ICD codes	6	2.23(1.41-3.52)	87.05	＜0.0001	7	1.84(1.38-2.45)	98.03	
Multiple metrics					1	2.11(1.16-3.84)	-	-
Questionnaire					1	1.27(0.94-1.71)	-	-
24h pH metrics					4	3.10(1.25-7.74)	59.39	0.0605
**Gender**								
male≤50%	2	1.59（0.65-3.88）	70.7	0.0647	1	1.65（1.20-2.27）	-	-
male＞50%	3	2.29（1.03-5.07）	82	0.0039	11	1.88（1.45-2.44）	96.87	＜0.0001
**Age**								
≤60	3	2.33（0.91-5.98）	79.2	0.0082	3	3.43(0.98-11.99)	95.74	＜0.0001
＞60	2	1.59（1.09-2.32）	30.32	0.2309	10	1.83(1.39,2.40)	97.1	＜0.0001
**Geographical region**								
North America	2	2.68（1.50-4.79）	86.38	0.0067	10	1.86(1.41-2.450	97.21	＜0.0001
Europe	1	1.30（0.82-2.06）	-	-	1	7.63(2.55,22.84)	-	-
Asia	3	2.33（0.91-5.98）	79.2	0.0082	4	2.03(1.48-2.77)	0	＜0.0001
**Number of participants**								
≤10000	6	2.23(1.41-3.52)	87.05	＜0.0001	11	1.89（1.44-2.46）	62.75	0.0028
＞10000					4	2.00（1.27-3.16）	98.95	＜0.0001
**Quality of study**								
Good	6	2.23(1.41-3.52)	87.05	＜0.0001	6	2.18(1.51-3.15)	85.54	＜0.0001
Fair					9	1.85(1.35-2.54)	96.04	＜0.0001

	Laryngeal cancer							
	n	RR(95%CI)	I^2(%)	*P*	n	OR(95%CI)	I^2(%)	*P*
Poor								
**Adjustments**								
BMI								
Yes	2	3.10（1.12-8.58）	83.67	0.0133	1	1.27(0.94-1.71)	-	-
No	4	1.93(1.09-3.39)	89.92	＜0.0001	14	2.06(1.61-2.64)	95.93	＜0.0001
**Alcohol consumption**								
Yes	3	2.29（1.03-5.07）	82	0.0039	6	1.62(1.19-2.22)	97.45	＜0.0001
No	3	2.19（1.11-4.33）	83.54	0.0023	9	2.42(1.80-3.25)	68.6	0.0013
**Smoking status**								
Yes	2	3.10（1.12-8.58）	83.67	0.0133	7	1.62(1.24-2.13)	96.95	＜0.0001
No	4	1.93（1.09-3.39）	89.92	＜0.0001	8	2.62(2.00-3.44)	53.91	0.0337
**Age**								
Yes	3	2.71（1.55-4.75）	67.88	0.0444	7	1.82(1.38-2.46)	97.98	＜0.0001
No	3	1.74（0.78-3.89）	92.36	＜0.0001	8	2.44(1.48-4.02)	69.52	0.0017
**Sex**								
Yes	3	2.71（1.55-4.75）	67.88	0.0444	7	1.82(1.38-2.46)	97.98	＜0.0001
No	3	1.74（0.78-3.89）	92.36	＜0.0001	8	2.44(1.48-4.02)	69.52	0.0017

Table S7. Meta-regression results

Covariate	Coefficient	SE	95%CI	z-value	p-value	Residual I^2
**Age**	0.869	0.4954	1.7539	0.0794	-0.1021	1.8400
**Region**	0.7156	0.2715	2.636	0.0084	0.1835	1.2476
**Number of participants**	0.6836	0.1626	4.2044	<.0001	0.3649	1.0022
**Sex**	1.3551	0.4308	3.1459	0.0017	0.5109	2.1994
**Smoking adjustments**	0.9829	0.1787	5.5005	<.0001	0.6327	1.3332
**Alcohol adjustments**	0.8971	0.1684	5.3263	<.0001	0.567	1.2272
**Age/sex adjustment**	0.8264	0.2121	3.8974	<.0001	0.4108	1.2421
**Definition of GERD**	1.0527	0.354	2.9734	0.0029	0.3588	1.7465

Figure S1. Funnel plot to assess publication bias across the studies evaluating the risk of GERD among subjects with laryngeal cancer

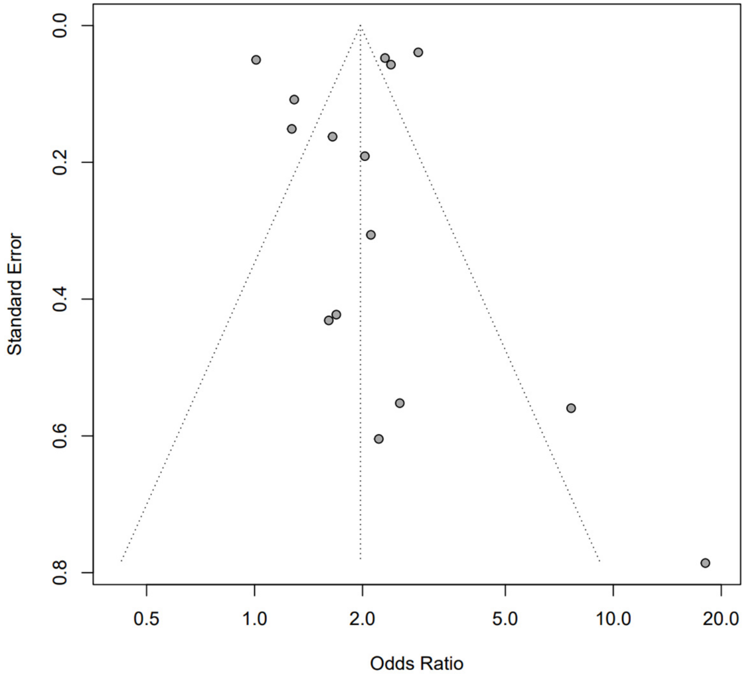

Material A. Study quality assessment for cohort studies (Newcastle-Ottawa Scale)

Study	Selection				Comparability	Outcome			Total no. of stars
	Exposed cohort	Nonexposed cohort	Ascertainment of exposure	Outcomes of interest		Assessment of outcome	Length of follow-up	Adequacy of follow-up	
Hsu, 2016 [15]	*	*	*	*	**	*	*	*	9
Hu, 2021 [51]	*	*	*	*	**	*	*	*	9
Kim, 2019 [8]	*	*	*	*	**	*	-	-	7
Kuo, 2015 [9]	*	*	*	*	**	*	*	*	9
Liao, 2024 [46]	*	*	-	*	**	-	*	*	7
Nilsson, 2005 [10]	*	*	*	*	*	*	*	-	7
Solaymani-Dodaran, 2004 [45]	*	-	*	*	**	*	*	*	8
Tran, 2023 [32]	*	*	-	*	**	-	*	*	7
Wang, 2021 [34]	*	*	*	*	**	-	*	*	8

-, no star(s) awarded.

Material B. Study quality assessment for case-control studies (Newcastle-Ottawa Scale)

Study	Selection				Comparability	Outcome			Total no. of stars
	Definition of cases	Representativeness of cases	Selection of controls	Definition of controls		Assessment of exposure	Method of ascertainment	Non-response rate	
Amarnath, 2022 [39]	*	*	-	*	**	*	*	*	8
Anis, 2018 [11]	-	*	-	*	**	*	*	-	6
Bacciu, 2004 [23]	*	*	-	*	**	*	*	*	8
Busch, 2015 [14]	*	*	*	*	**	-	*	*	8
Choi, 2019 [24]	*	*	*	*	**	-	*	-	7
Dagli, 2004 [25]	*	-	-	*	-	*	*	-	4
Doustmohammadian, 2011 [26]	*	*	-	*	**	*	*	-	7
Dresler, 2017 [50]	-	-	-	-	*	-	*	-	2
Duran, 2008 [47]	*	*	-	-	**	*	*	-	6
El-Serag, 2001 [27]	-	*	-	*	**	-	*	-	5
Francis, 2010 [12]	-	*	-	*	**	-	*	-	5
Galli, 2002 [28]	-	*	-	*	**	*	*	-	6
Ghanem, 2007 [48]	*	-	-	-	**	*	*	-	5
Hayasaka, 2024 [49]	*	*	-	*	**	*	*	-	7
Kim, 2019 [8]	-	*	*	*	**	-	*	*	7
Koufman, 1991 [29]	-	*	-	*	*	*	*	-	5
Wu, 2003 [44]	*	*	*	*	**	-	*	*	8
Ozlugedik, 2005 [30]	-	-	-	*	*	*	*	*	5
Parsel, 2020 [31]	-	*	-	*	**	-	*	*	6
Riley, 2017 [13]	-	*	*	*	**	-	*	*	7
Vaezi, 2006 [33]	*	*	-	*	**	-	*	*	7

-, no star(s) awarded.

PRISMA Checklist

Section and Topic	Item	Checklist item	Location where item is reported
**TITLE**			Page 1
Title	1	Identify the report as a systematic review.	
**ABSTRACT**			Page 1
Abstract	2	See the PRISMA 2020 for Abstracts checklist.	
**INTRODUCTION**			
Rationale	3	Describe the rationale for the review in the context of existing knowledge.	Page 2
Objectives	4	Provide an explicit statement of the objective(s) or question(s) the review addresses.	Page 2
**METHODS**			
Eligibility criteria	5	Specify the inclusion and exclusion criteria for the review and how studies were grouped for the syntheses.	Page 3
Information sources	6	Specify all databases, registers, websites, organisations, reference lists and other sources searched or consulted to identify studies. Specify the date when each source was last searched or consulted.	Page 3
Search strategy	7	Present the full search strategies for all databases, registers and websites, including any filters and limits used.	
Selection process	8	Specify the methods used to decide whether a study met the inclusion criteria of the review, including how many reviewers screened each record and each report retrieved, whether they worked independently, and if applicable, details of automation tools used in the process.	Page 3
Data collection process	9	Specify the methods used to collect data from reports, including how many reviewers collected data from each report, whether they worked independently, any processes for obtaining or confirming data from study investigators, and if applicable, details of automation tools used in the process.	Page 3
Data items	10a	List and define all outcomes for which data were sought. Specify whether all results that were compatible with each outcome domain in each study were sought (e.g. for all measures, time points, analyses), and if not, the methods used to decide which results to collect.	Page 3
	10b	List and define all other variables for which data were sought (e.g. participant and intervention characteristics, funding sources). Describe any assumptions made about any missing or unclear information.	Page 3
Study risk of bias assessment	11	Specify the methods used to assess risk of bias in the included studies, including details of the tool(s) used, how many reviewers assessed each study and whether they worked independently, and if applicable, details of automation tools used in the process.	Page 3

Section and Topic	Item	Checklist item	Location where item is reported
Effect measures	12	Specify for each outcome the effect measure(s) (e.g. risk ratio, mean difference) used in the synthesis or presentation of results.	Page 3,4
Synthesis methods	13a	Describe the processes used to decide which studies were eligible for each synthesis (e.g. tabulating the study intervention characteristics and comparing against the planned groups for each synthesis (item #5)).	Page 3,4
	13b	Describe any methods required to prepare the data for presentation or synthesis, such as handling of missing summary statistics, or data conversions.	Page 3,4
	13c	Describe any methods used to tabulate or visually display results of individual studies and syntheses.	Page 3,4
	13d	Describe any methods used to synthesize results and provide a rationale for the choice(s). If meta-analysis was performed, describe the model(s), method(s) to identify the presence and extent of statistical heterogeneity, and software package(s) used.	Page 3,4
	13e	Describe any methods used to explore possible causes of heterogeneity among study results (e.g. subgroup analysis, meta-regression).	Page 3,4
	13f	Describe any sensitivity analyses conducted to assess robustness of the synthesized results.	Page 3,4
Reporting bias assessment	14	Describe any methods used to assess risk of bias due to missing results in a synthesis (arising from reporting biases).	Page 3,4
Certainty assessment	15	Describe any methods used to assess certainty (or confidence) in the body of evidence for an outcome.	Page 3,4
**RESULTS**			
Study selection	16a	Describe the results of the search and selection process, from the number of records identified in the search to the number of studies included in the review, ideally using a flow diagram.	Page 4
	16b	Cite studies that might appear to meet the inclusion criteria, but which were excluded, and explain why they were excluded.	Table S1
Study characteristics	17	Cite each included study and present its characteristics.	Table 1
Risk of bias in studies	18	Present assessments of risk of bias for each included study.	Table 1, Material A and B
Results of individual studies	19	For all outcomes, present, for each study: (a) summary statistics for each group (where appropriate) and (b) an effect estimate and its precision (e.g. confidence/credible interval), ideally using structured tables or plots.	Figure 2, 3, and 4
Results of syntheses	20a	For each synthesis, briefly summarise the characteristics and risk of bias among contributing studies.	Page 6-10
	20b	Present results of all statistical syntheses conducted. If meta-analysis was done, present for each the summary estimate and its	Page 6-10

Section and Topic	Item	Checklist item	Location where item is reported
		precision (e.g. confidence/credible interval) and measures of statistical heterogeneity. If comparing groups, describe the direction of the effect.	
	20c	Present results of all investigations of possible causes of heterogeneity among study results.	Page 6-10
	20d	Present results of all sensitivity analyses conducted to assess the robustness of the synthesized results.	Page 6-10
Reporting biases	21	Present assessments of risk of bias due to missing results (arising from reporting biases) for each synthesis assessed.	Page 6-10
Certainty of evidence	22	Present assessments of certainty (or confidence) in the body of evidence for each outcome assessed.	Page 6-10
**DISCUSSION**			
Discussion	23a	Provide a general interpretation of the results in the context of other evidence.	Page 10-12
	23b	Discuss any limitations of the evidence included in the review.	Page 11
	23c	Discuss any limitations of the review processes used.	Page 11
	23d	Discuss implications of the results for practice, policy, and future research.	Page 11-12
**OTHER INFORMATION**			
Registration and protocol	24a	Provide registration information for the review, including register name and registration number, or state that the review was not registered.	Page 2
	24b	Indicate where the review protocol can be accessed, or state that a protocol was not prepared.	Page 2
	24c	Describe and explain any amendments to information provided at registration or in the protocol.	Not applicable
Support	25	Describe sources of financial or non-financial support for the review, and the role of the funders or sponsors in the review.	Page 12
Competing interests	26	Declare any competing interests of review authors.	Page 12
Availability of data, code and other materials	27	Report which of the following are publicly available and where they can be found: template data collection forms; data extracted from included studies; data used for all analyses; analytic code; any other materials used in the review.	Table 12

From: Page MJ, McKenzie JE, Bossuyt PM, Boutron I, Hoffmann TC, Mulrow CD, et al. The PRISMA 2020 statement: an updated guideline for reporting systematic reviews. BMJ 2021;372:n71. doi: 10.1136/bmj.n71. This work is licensed under CC BY 4.0. To view a copy of this license, visit https://creativecommons.org/licenses/by/4.0/
